# From pre-existing vulnerabilities to daily stressors, what is the impact of climate change on mental health and gender inequity in Asia region?

**DOI:** 10.1192/j.eurpsy.2025.283

**Published:** 2025-08-26

**Authors:** V. Kaya, L. Clouin, T. Iglesias Zayas, K. Le Roch, R. Alessandri, E. Dozio

**Affiliations:** 1 Operations; 2 Mental Health and Psychosocial Support; 3 Action contre la faim, Montreuil, France

## Abstract

**Introduction: Background:**

While climate change affects millions of people in South and Southeast Asia, women and girls are disproportionately impacted, largely due to pre-existing vulnerabilities given their traditional gender roles and intensified adaptive capacity and sensitivity to climate change. They have often less representation and decision-making power in governance processes and structures involved in the development and implementation of climate change adaptation and mitigation policies. Moreover, they face mental health and psychosocial problems due to various climate-related stressors.

**Objectives:**

The study aimed at exploring the perceptions of people living in contexts affected by climate change in order to better understand the impact on the psychosocial conditions of women and girls.

**Methods:**

In 2023, online and in situ interviews were conducted with 30 individuals (15 women, 15 men) from the government, international and national organisations as well as academic and research institutions in Afghanistan, Bangladesh, Myanmar, Nepal, and Pakistan. Moreover, eight focus group discussions were conducted in Bangladesh (Kurigram and Sathkira districts) and Nepal (Rasuwa and Udayapur districts) with 71 community members, including 40 women and girls (aged 14 to 54 years old) and 31 men and boys (aged 15 to 70 years old).

**Results:**

In Bangladesh, both districts face extreme weather like drought and floods, which leads to the migration of men. An increased involvement of women in farming, alongside their household responsibilities. This has transformed the family dynamic and women, as household head, have become the primary decision-makers. In Nepal, there has been a lack of awareness among women and men on the impact of climate change on their living conditions. The significant shift in the traditional gender divisions of labour has not brought an equitable transfer of assets and resources that could help women to cope after a climate-related event. Ultimately, women suffer from mental health issues.

**Conclusions:**

This study shows some causal links between climate change and the psychosocial conditions of women and girls which confirm the necessity to develop gender-responsive climate change strategies, to improve access to mental health services and to prevent long-term changes within communities.

**Disclosure of Interest:**

None Declared

